# Sustainable Extraction of Antioxidant Phytocompounds from Yellow Onion Wastes for Value-Added Product Development

**DOI:** 10.3390/antiox15050632

**Published:** 2026-05-15

**Authors:** Anca M. Rosca, Adina I. Gavrila, Ioan Calinescu, Christina Zalaru, Mihaela D. Popescu, Alexandra Ene-Manea, Justinian A. Tomescu

**Affiliations:** 1 Faculty of Chemical Engineering and Biotechnologies, National University of Science and Technology Politehnica Bucharest, 011061 Bucharest, Romania; anca_maria.rosca@stud.fim.upb.ro (A.M.R.); ioan.calinescu@upb.ro (I.C.); mihadana2001@gmail.com (M.D.P.); 2 Faculty of Chemistry, University of Bucharest, 050663 Bucharest, Romania; chmzalaru@gmail.com; 3 HOFIGAL Export-Import S.A., 042124 Bucharest, Romania; manea_alexa96@yahoo.com; 4 Faculty of Agriculture, Department of Biology, University of Agronomic Sciences and Veterinary Medicine of Bucharest, 011464 Bucharest, Romania

**Keywords:** green extraction, ultrasounds, yellow onion peels, antioxidants, optimization

## Abstract

Yellow onion (*Allium cepa* L.) outer skins are a high-volume agricultural waste that can be converted into commercially valuable bioproducts using various extraction techniques. This research focused on optimizing a green ultrasound-assisted extraction (UAE) method which allows for the isolation of several phytochemicals valued for their health benefits, such as polyphenols and flavonoids. HPLC/UV analysis of the extracts showed that the main component was quercetin. A one-factor-at-a-time (OFAT) design was used to identify the extraction parameters needed in order to maximize the amount of extracted target phytochemicals. The polyphenols, flavonoids and quercetin contents, along with the antioxidant activity of the extracts, were optimized by response surface methodology using a Box–Behnken design. Ultrasound amplitude, ethanol concentration, and time were selected as the most appropriate variables. The final results showed that TPC ranged from 78.16 to 97.16 mg GAE/g DM, TFC ranged from 22.77 to 26.46 mg QE/g DM, while CUPRAC values varied between 145.24 and 163.75 mg TE/g DM. The optimal extraction conditions were determined using a Box–Behnken model as 30% ultrasound amplitude, 53% ethanol concentration, and an extraction time of 13 min. The use of these conditions allowed the TPC, TFC and CUPRAC to show predicted values of 97.8 mg GAE/g DM, 27.2 mg QE/g DM, and 159.8 mg TE/g DM, respectively. These findings indicate that onion skin extracts could represent a green and promising source of antioxidant phytochemicals.

## 1. Introduction

Onion (*Allium cepa* L.) is one of the most economically important vegetable crops worldwide, the second most cultivated behind tomatoes, with the raw fresh bulbs being valued both in gastronomy and in phytotherapy, due to their numerous proved biological effects [1]. Processed onion is also very popular, with dried flakes and powders being in especially high demand [2]. However, large-scale processing also implies the production of proportionately high amounts of waste, posing significant issues regarding its proper disposal. Onion solid waste (OSW) accounts for around 550,000 tons per year or roughly 38% of the total world onion production [1]. OSW is the result of industrial onion processing and post-harvest losses, being made up of the inedible peels or colored outer skins, the two outer fleshy scales, onion roots, the apical components and bulbs deemed unfit for human consumption [2]. Therefore, newer and more efficient waste management strategies are needed, such as the development of value-added products [3].

Onions are rich in phytochemicals which can be easily used in food or nutraceutical applications, with the inedible outer skins containing considerably more bioactive compounds than the edible flesh [1]. The overall phytochemical profile of brown onion skin is composed of up to 5.3% polyphenols [3,4], with the most substantially represented class being flavonoids [5]. However, the general aim of nutraceutical applications is not the use of whole or crude extracts but rather the use of single-component ones, as is the case of quercetin, one of the most important flavonols in onion peels [6]. With regard to quantification, colored onion varieties have been shown to contain quercetin at levels up to 14 times greater than those found in garlic and roughly twice those measured in white onion varieties [1].

Extraction is one of the most important steps in investigating onion peel polyphenols and their applications as value-added products by isolating the targeted compounds through the use of various procedures and solvents [7]. Conventional extraction methods are techniques such as maceration, Soxhlet extraction, digestion and percolation [8,9,10]. These methods are generally associated with a series of disadvantages, including long extraction times, use of expensive and toxic solvents, issues regarding solvent evaporation and other post-processing steps, and limited selectivity for target compounds [7,11,12,13]. Therefore, in order to overcome these limitations, both green extraction and non-conventional techniques have gained significant popularity.

Although a variety of advanced extraction methods are currently available, ultrasound-assisted extraction (UAE) is considered one of the most cost-effective and widely applied modern techniques, due to its relatively low operational cost, scalability, and ability to balance efficiency with reduced environmental impact [7,14]. In contrast to conventional techniques which rely heavily on heat transfer, solvent use and passive diffusion, UAE offers several advantages such as shorter extraction times, lower solvent consumption, improved mass transfer, and better recovery of bioactive compounds achieved under milder operating conditions. These benefits are mainly attributed to acoustic cavitation, which generates microbubbles that collapse near the plant matrix, producing microjets and local shear forces. As a result, the plant cell structure is disrupted, solvent penetration is enhanced, and the diffusion of target compounds into the extraction medium is accelerated [8,15]. However, UAE also presents some limitations regarding extraction parameters such as ultrasound power, extraction time, solvent composition, and temperature, issues that may lead to unnecessary energy consumption or possible degradation of thermosensitive compounds [16]. Therefore, process optimization needs to be implemented, especially regarding efficient recovery of antioxidant compounds from renewable resources, such as agricultural waste.

Response surface methodology (RSM) is a multivariate statistical tool used to model an experimental domain through a response function, accordingly a predefined theoretical design [17]. As an empirical modeling technique, its purpose is to define the relationship between a set of controlled experimental factors and the observed outcomes [18].

Several studies have explored the recovery of bioactive compounds from onion by-products; however, many of them have focused mainly on red onion skins or scales [19,20,21,22]. Red onion peels are generally richer in pigmented compounds, especially anthocyanins, whereas yellow onion peels are mainly recognized as a source of flavonols such as quercetin and its derivatives [23]. Therefore, extraction conditions optimized for red onion by-products cannot be directly extrapolated to yellow onion peels, supporting the need for a specific optimization study focused on this matrix.

The main objective of this experimental study was an integrated evaluation and optimization of UAE from yellow onion (*Allium cepa* L.) peels by considering multiple phytochemical responses, antioxidant activities, quercetin content, and energy consumption. The novelty of this study lies in the systematic optimization approach using the Box–Behnken design (BBD) under response surface methodology (RSM) to identify the optimal extraction parameters from onion peels, an underutilized agro-industrial by-product, thereby contributing to the valorization of plant waste as a sustainable source of compounds with biological activities. Moreover, the energy consumption associated with different UAE conditions and its comparison with conventional extraction methods remain insufficiently discussed. To the best of our knowledge, the simultaneous optimization of these responses using UAE and Box–Behnken design for yellow onion peels has not been previously reported.

## 2. Materials and Methods

### 2.1. Materials

Inedible yellow onion (*Allium cepa* L.) peels were obtained from a local market (Bucharest, Romania). The onion skins were dried at 50 °C using an air flow heating oven (Force Air Drying Oven OVF, LBX instruments, LabBox, Barcelona, Spain), milled using an electric grinder and screened to a particle size less than 0.5 mm. Prepared vegetal material was dosed, sealed in glass vessels, and stored at room temperature away from light, heat, and humidity. The residual moisture of the onion peel powder was determined using a thermogravimetric balance (PMB 202 Moisture Analyzer, Adam Equipment Co., Ltd., Bletchley, UK) and was found to be 10.45% (w). Subsequently, all the results in this work are presented as per gram of dry matter (g DM).

The standards for determining onion skin powder biocompounds (polyphenols, flavonoids) and antioxidant activity were gallic acid, quercetin, and Trolox, respectively, purchased from Sigma-Aldrich Co, Bucharest, Romania. Ethanol (96%), copper chloride, neocuproine, ammonium acetate, Folin–Ciocalteu reagent, sodium carbonate, anhydrous aluminum chloride, sodium acetate, and 2,2-diphenyl-1-picrylhydrazyl (DPPH) were also purchased from Sigma-Aldrich Co, Bucharest, Romania. All reagents used for colorimetric analyses were of analytical grade.

The standard used for HPLC analysis and quercetin quantification was quercetin aglycone, purchased from Sigma-Aldrich Co, Bucharest, Romania. Methanol, acetonitrile, formic acid, and water were also purchased from Sigma-Aldrich Co, Bucharest, Romania. All reagents used for this analysis were of HPLC grade.

### 2.2. Ultrasound-Assisted Extraction (UAE) Procedure

The extraction of bioactive compounds from onion peel powder was performed using a batch system consisting of a jacketed glass reactor. To maintain the optimal temperature, water was continuously circulated between the reactor jacket and a thermostat throughout the entire extraction process. Ultrasonication was applied with a Vibracell VCX750 (Sonics & Materials, Inc.; Newtown, CT, USA) ultrasonic probe, which was inserted directly into the extraction mixture within the reactor. The extraction parameters were varied according to the experimental design presented below. For comparison, conventional extraction was carried out using a magnetic stirring system, set to 900 rpm, in place of the ultrasonic probe. Following extraction, the plant material was separated by centrifugation at 2500 rpm for 5 min. The supernatant was kept in a freezer until subsequent HPLC analysis and further assays for bioactive compound quantification. All experiments were performed in duplicate and bioactive compound analysis in triplicate.

### 2.3. Phytocompound Analysis

#### 2.3.1. Total Phenolics Content (TPC)

TPC was determined colorimetrically using the Folin–Ciocalteu assay (ISO 14502-1 standard [24]) with minor modifications, as described by Asofiei et al. [25]. Briefly, 0.5 mL of diluted sample was mixed with 5 mL of 10% Folin–Ciocalteu reagent and stirred for 5 min in order for the reaction to occur. Then, 1.5 mL of 20% Na_2_CO_3_·10H_2_O and 3 mL of distilled water were added. Finally, the samples were left to stand in the dark, at room temperature, for 60 min. The absorbance was measured at 760 nm using a UV mini-1240 UV-Visible Scanning spectrophotometer (Shimadzu Corporation, Kyoto, Japan). Final results were expressed as milligrams of gallic acid equivalents per gram of dry matter (mg GAE/g DM) using a standard GA solution (1–5 mg/mL) curve.

#### 2.3.2. Total Flavonoid Content (TFC)

TFC was determined colorimetrically using the aluminum chloride assay, as described by Shraim et al. [26] and by Oboh et al. [27], with minor modifications. Briefly, 1 mL of appropriately diluted sample was mixed with 3 mL ethanol, 0.2 mL 10% AlCl_3_, 0.2 mL 1 M sodium acetate, and 5.6 mL distilled water. The mixture was incubated in the dark, at room temperature, for 40 min. The absorbance of the reaction mixture was subsequently measured at 425 nm, with quercetin being used as a reference standard. Results were expressed as milligrams of quercetin equivalents per gram of dry matter (mg QE/g DM) using a standard quercetin solution (25–300 mg/mL) curve.

### 2.4. Antioxidant Activity

#### 2.4.1. CUPRAC Assay

The antioxidant activity of the extracts was determined using the cupric reducing antioxidant capacity (CUPRAC) method, as described by Gavrila et al. [28] with slight modifications. Briefly, 1 mL 0.01 M CuCl_2_ aqueous solution, 1 mL 0.0075 M neocuproine ethanolic solution, 1 mL ammonium acetate buffer solution, and an aliquot of 1.1 mL appropriately diluted extract were mixed thoroughly in a vial and allowed to stand in the dark, at room temperature, for 30 min. The absorbance was measured at 450 nm. The results were expressed as milligrams of Trolox equivalents per gram of dry matter (mg TE/g DM), using a standard curve which corresponds to 0–0.25 mg/mL Trolox for quantification.

#### 2.4.2. DPPH Radical Scavenging Assay (RSA)

The RSA scavenging activity of the extracts was evaluated by using the DPPH assay, a form of colorimetric stable free radical scavenging assay whose mechanism was first described by Blois [29] and Xiao et al. [30], with slight modifications. First, 0.1 mL appropriately diluted sample was mixed with 3 mL 0.004% DPPH ethanolic solution and left to stand in the dark, at room temperature, for 30 min. The absorbance was measured at 517 nm. Results were expressed as radical scavenging activity (% RSA) and half-maximal inhibitory concentration (IC50) following the procedure described by Xiao et al. [30]. RSA was determined using Equation (1), where *A_blank_* is the absorbance of a blank sample prepared by substituting the sample volume with ethanol and *A_sample_* is the absorbance of the sample containing the analyte.

(1) $$\%\;RSA=\frac{A_{blank}-A_{sample}}{A_{blank}}\times 100$$

### 2.5. HPLC Analysis

Quercetin qualitative and quantitative analysis was performed via RP-HPLC-DAD analysis using the method developed by Dangnon et al. [31], with slight modifications. HPLC analysis was achieved using a Hitachi Chromaster HPLC system (Hitachi High-Tech, Tokyo, Japan), equipped with the following modules: 5160 pump, 5310 column oven, 5260 thermostat autosampler, and 5430 DAD detector. Separation was performed on a ZORBAX SB-C18 4.6 × 150 mm, 3.5 µm column (ZORBAX^®^ Agilent Technologies, Inc. Santa Clara, CA, USA). The mobile phase consisted of a mixture of acetonitrile and methanol in a 1:1 ratio with 1% formic acid (A) and water with 1% formic acid (B). The gradient elution was carried out at 1 mL/min, as follows: 0 min: 10% A–90% B; 5 min: 30% A–70% B; 20 min: 40% A–60% B; 25 min: 42.5% A–57.5% B; 26 min: 10% A–90% B; 30 min: 10% A–90% B. Standard stock solutions were prepared by dissolving a quercetin reference standard in methanol, with concentrations ranging from 24 µg/mL to 193 µg/mL. The quercetin content was determined using a standard quercetin calibration curve, with absorbance measured at a fixed wavelength of 369 nm. The HPLC chromatogram of the quercetin standard showed a peak at a retention time of 19.8 min, while the optimized extract showed a corresponding quercetin peak at the same retention time, supporting the presence of quercetin in the extract (Figures S1 and S2). An additional intense peak was observed at approximately 13 min in the extract chromatogram; however, this compound could not be unambiguously identified under the present HPLC/UV conditions, as the method was developed for targeted quercetin quantification and no corresponding authentic standard or LC-MS/MS data were available. The calibration curve exhibited good linearity over the investigated concentration range, with a strong correlation coefficient (R^2^ = 0.99998), and was expressed by the equation y = 18.78298x − 5.45909, where y represents the peak area and x represents the concentration. All samples and standards were diluted with methanol, filtered through a 0.2 µm PTFE filter, and 5 µL of each solution was injected into the HPLC system. Data acquisition was performed at a wavelength of 369 nm.

### 2.6. Statistical Analysis and Experimental Design

The experimental program was performed in triplicate (*n* = 3) and data were reported as mean value ± standard deviation (SD). Statistical analysis was conducted using XLSTAT Version 2019.1 (Addinsoft, New York, NY, USA). ANOVA was employed to assess the variations of the obtained data and the differences were considered statistically significant for *p* < 0.05. Specifically, Duncan’s test was applied to compare means, with statistical significance established for the outcomes. One factor at a time (OFAT) was used to evaluate the factor levels for choosing the significant parameters.

The Design-Expert v11 software (Stat-Ease Inc., Minneapolis, MN, USA) was employed to predict the optimal settings that yielded the highest desirability value for each response. According to the OFAT study the critical variables identified were ethanol concentration (X_1_), ultrasound amplitude (X_2_), and extraction time (X_3_). Their levels were defined as low (−), medium (0), and high (+), and the study limits and codes are presented in Table 1.

The experimental data generated by the Box–Behnken design (BBD) served as the basis for determining optimal conditions using the predicted model. Furthermore, the software generated predicted values for these responses, which were then validated against actual experimental data to confirm the model’s accuracy and reliability.

## 3. Results and Discussion

The extraction process of bioactive compounds from yellow onion peels was studied in two distinct experimental stages. The first stage involved a parametric study, using the OFAT technique to simplify the evaluation of factor levels in order to choose the significant parameters, followed by a second stage where the process was optimized using a Box–Behnken experimental design.

### 3.1. Effect of Extraction Parameters on Bioactive Compound Content Using the OFAT Technique

#### 3.1.1. Effect of Ultrasound Power on the Extraction of Bioactive Compounds from Yellow Onion Peels

The critical processing factor determining the efficiency of the sonochemical-assisted extraction is the ultrasound power delivered into the system. Monitoring acoustic power is essential for determining the efficiency of energy transfer within the sonicated liquid. The enhanced efficiency of UAE can be attributed to its extraction mechanism that involves cavitation within the heterogeneous solvent–plant mixture. The asymmetrical collapse of the resulting bubbles produces intense, localized microjets of solvent that impinge upon the solid material, facilitating the release of intracellular compounds. Specifically, the application of ultrasound generates both a grinding effect and a mixing effect. These phenomena contribute to the increased yield by reducing the particle size of the plant matrix and by promoting superior contact between the solid material and the solvent [15].

In order to evaluate the effect of ultrasound power in the UAE, the ultrasound amplitudes varied from 20 to 60%, and the effect of duty cycle was probed by applying ultrasounds in pulses (5 s on/5 s off). The constant extraction conditions were ethanol concentration 60%, extraction time 10 min, temperature 50 °C, and solvent-to-plant ratio 20 mL/g. Table 2 presents the effect of ultrasound power and duty cycle on the extraction of bioactive compounds and antioxidant activity.

To investigate the intensification effect of ultrasounds on the recovery of bioactive antioxidant compounds from yellow onion peels, a comparative conventional extraction was performed (Table 2, entry 1). Conventional extraction was carried out under identical extraction conditions replacing the ultrasonic probe by a magnetic stirring system (stirring rate 900 rpm).

The sonication duty cycle and ultrasound-specific power exert a significant influence on the quantity of bioactive products extracted from onion peels. The one-way ANOVA revealed that, by increasing the specific ultrasonic power from 0 to 20.14 W/gDM, the bioactive compound content and antioxidant activity increase significantly (*p* < 0.05). The best results were obtained at a 30% amplitude. When increasing the amplitude from 30% to 60% a significant decrease in TPC, TFC, and antioxidant activity was observed. The data further demonstrate that continuous sonication yields significantly superior outcomes compared to pulsed sonication (Table 2), resulting in a higher extracted quantity of polyphenols and flavonoids. However, the data obtained are correlated with CUPRAC antioxidant activity, DPPH radical scavenging activity (RSA) and IC50, which were significantly higher for continuous operation compared to the pulsed mode. The results indicate that UAE led to significantly higher amounts of bioactive compounds (*p* < 0.05) compared to conventional extraction (Table 2). These outcomes demonstrate a positive correlation with one another. UAE facilitates the extraction of a greater quantity of total phenolic compounds, and, consequently, flavonoids. Furthermore, the CUPRAC antioxidant activity correlates well with RSA and with IC50.

In addition, HPLC quantification of quercetin (Table 2) confirmed a direct correlation between extraction parameters and yield. Continuous sonication facilitated the recovery of a greater amount of quercetin compared to pulsed operation. Notably, the analysis also indicates a lower quercetin yield at the highest amplitude tested (60%), suggesting potential degradation phenomena at this intensity [15]. Consequently, based on the collective data from bioactive compound analysis and HPLC results, it is appropriate to perform quercetin extraction using continuous sonication at lower amplitude levels. UAE enabled the recovery of 6.3 mg quercetin/g DM whereas conventional extraction yielded only 5.5 mg quercetin/g DM. Other studies using UAE for the extraction of quercetin and its derivatives from onion skin waste have reported that ultrasound power was the most effective factor on the extraction of quercetin. For example, Jin et al. [32] found that increasing ultrasonic power improved quercetin recovery by approximately 10%, reaching 4.09 ± 0.29 mg QE/g under the optimized extraction conditions. According to these results, the ultrasonic power had a significant effect on the extraction process and an ultrasound amplitude range of 20–40% was selected for the BBD, and 30% amplitude was used for subsequent extractions.

#### 3.1.2. Effect of Solvent Concentration

An essential parameter for antioxidant extraction is the solvent. Most bioactive compounds with antioxidant properties, as polyphenols and flavonoids, are easily extracted from plants by polar organic solvents in aqueous solutions [33]. Quercetin in its aglycone form exhibits solubility in a relatively narrow range of solvents. Under standard conditions, quercetin aglycone is highly soluble in methanol, soluble in ethanol, and nearly insoluble in water. In contrast, glycosylated quercetin derivatives are also soluble in alcohol but, unlike the aglycone, are partially soluble in water [34]. Consequently, the optimal selection of a specific solvent or the formulation of a solvent mixture for selective extraction plays a crucial role in achieving a high extraction yield [35]. The low toxicity and economic accessibility of hydroethanolic solutions make them a common choice for extraction of plant-based antioxidants. Therefore, the effect of solvent on bioactive compounds was studied using various concentrations of ethanol (0–100% *v*/*v*), while other extraction conditions were kept constant as ultrasonic amplitude 30%, solvent-to-plant ratio 20 mL/g, extraction time 10 min, and temperature 50 °C.

As shown in Table 3, the one-way ANOVA indicated that TPC, TFC, CUPRAC, and RSA increased significantly (*p* < 0.05) when the ethanol concentration changed from 20% to 60%. Based on the principle of miscibility and similarity, extraction efficiency is maximized when solvent polarity is similar to that of plant solutes. As antioxidant activity reached its maximum at an ethanol concentration of 60%, the solvent polarity was close to bioactive compound polarity [36]. The extreme concentrations of 0% (water) and 100% (ethanol) yielded the poorest outcomes, attributable to the low solubility of quercetin in either solvent alone and the reduced extraction efficiency for other bioactive compounds [35]. However, TPC, TFC, and antioxidant activity demonstrated superior values at the 60% ethanol concentration. Other studies investigated the solvent effect on UAE extraction. For instance, Benito-Román et al. [37] reported that aqueous ethanol is commonly preferred for extracting quercetin from onion skin waste in the range of 50–75% for human-consumption-related applications, mainly due to safety considerations. They also found that 70% ethanol gave high TPC, TFC, and antioxidant activity in onion skin waste extraction [37].

Correlating these findings led to the selection of 60% (*v*/*v*) ethanol as the most effective solvent concentration for this matrix. This conclusion is further supported by HPLC analysis (Table 3), which identifies the sample prepared with 60% ethanol as having the highest quercetin content. Based on these outcomes, the ethanol concentration range of 40–80% was selected for the BBD, and 60% ethanol in water concentration was used for the following single-factor tests.

#### 3.1.3. Effect of Plant-to-Solvent Ratio

The influence of the solvent-to-plant (S/P) ratio on extraction processes is complex, stemming from the fundamental mechanisms of mass transfer. This parameter governs plant particle swelling, solvent penetration, solute solubilization, and the subsequent diffusion of target compounds into the bulk solvent [15]. The influence of S/P ratio on the bioactive compound content and antioxidant capacities of the extract from onion peels were explored at three levels ranging from 10:1 to 30:1 mL/g, with other conditions maintained as follows: ultrasound amplitude, 30%; ethanol concentration, 60% (*v*/*v*); extraction time, 10 min; and temperature, 50 °C (Table 4). 

As can be seen in Table 4, the total polyphenol and flavonoid contents and CUPRAC antioxidant activity and IC50 were enhanced with the increase in the S/P ratio from 10:1 to 20:1 mL/g and significantly decreased when the S/P ratio was higher than 20:1 mL/g. A possible explanation is that, while a larger solvent volume intuitively facilitates the extraction of a greater absolute quantity of bioactive compounds, a lower volume enhances selectivity: components that are more soluble are extracted first and saturate the solution, potentially leaving less soluble compounds behind. However, a lower limit for the solvent quantity exists, below which only swelling processes predominate, and effective extraction is negligible [38]. When the S/P ratio reached 20:1 (mL/g), the equilibrium of the dissolution process was reached.

However, the HPLC analysis for quantification of quercetin (Table 4) exhibited a strong correlation specifically with CUPRAC antioxidant activity. This indicates that, while the extracts are active, this activity cannot be attributed solely to quercetin, which was extracted in lower quantities at a 20:1 (mL/g) ratio. Instead, it is likely due to a combination of quercetin and other antioxidant polyphenolic compounds present in the extracts. Additional considerations for selecting the S/P ratio involve the energy demands of both the extraction and subsequent downstream processing. In alignment with green chemistry principles [39], these combined processes should be designed to minimize energy consumption while maximizing the yield of target products. Specifically, the use of an excessive solvent volume increases the energy required for extract concentration and solvent recovery. These outcomes agree with those found by Hammad et al. [20], who found that S/P ratios from 10:1 (mL/g) to 30:1 (mL/g) were used for the polyphenol and flavonoid extraction from onion solid waste and a further increase in this parameter decreased the yield of extracted compounds. As an S/P ratio of 20–30% provided a small difference in the yield of the extraction process, a 20:1 mL/g ratio was used for the next extractions and the S/P ratio factor was not included in the BBD for further optimization.

#### 3.1.4. Effect of Temperature

Temperature plays a critical role in extraction efficiency, particularly regarding bioactive compounds derived from plant biomass. Most secondary metabolites in plants are thermolabile, making them susceptible to thermal degradation at elevated temperatures. Consequently, conventional extraction methods—often characterized by significant thermal fluctuations—are increasingly replaced by non-conventional techniques. However, the latter are not entirely exempt from thermal management challenges; extraction mechanisms can generate heat either throughout the bulk medium or via localized “hot-spots” [15]. Flavonoids, including quercetin, typically maintain stability up to approximately 70 °C. Generally, aglycone forms exhibit higher thermal stability than their glycosylated counterparts, with degradation primarily occurring through oxidation, hydrolysis, isomerization, and polymerization [40]. Furthermore, the choice of solvent significantly influences stability; ethanol and aqueous-ethanol mixtures provide a stabilizing effect, whereas pure water often accelerates degradation [15,40]. To study the effect of temperature on polyphenol and flavonoid extraction, different temperatures (40–70 °C) were evaluated, while other factors were fixed: ultrasound amplitude 30%, ethanol concentration 60%, extraction time 10 min, S/P ratio 20:1 (mL/g), and the results are presented in Table 5.

As shown in Table 5, TPC, TFC, CUPRAC antioxidant activity, and RSA of the extract of yellow onion peels increased when the temperature increased from 40 to 60 °C. This was due to the fact that higher temperatures can reduce solvent viscosity, enhance molecular motion, and increase solubility [15]. The experimental results in this study align with existing literature: extraction at 40 °C yielded a low concentration of antioxidant compounds. However, significant variations were observed in flavonoid concentration, antioxidant activity, and DPPH radical inhibition. HPLC analysis (Table 5) confirmed that the maximum yield of quercetin was achieved at 50 °C. However, when the temperature was higher than 60 °C, a decline in bioactive content was observed.

The obtained outcomes are consistent with those previously reported by Jang et al. [41], who found that temperature, together with ethanol concentration, was among the most influential parameters for quercetin extraction from onion solid waste with optimum conditions of 59% ethanol and 49 °C. The observed effect was due to the fact that higher temperatures may induce antioxidant degradation. Correlating these findings, it can be concluded that 50 °C represents the optimal temperature for the quantitative extraction of antioxidant compounds from onion peels.

#### 3.1.5. Effect of Extraction Time

In sonochemistry and ultrasound-assisted extraction (UAE), extraction time is a critical parameter that directly governs the yield of bioactive compounds. Insufficient sonication intervals result in incomplete recovery of the target analytes. Conversely, prolonged exposure to ultrasound is detrimental, as it facilitates the degradation of sensitive compounds. The mechanisms underlying this degradation are primarily attributed to the rise in temperature within the extraction medium and the sonolysis of water molecules. The latter involves the homolytic cleavage of water, leading to the formation of highly reactive free radicals and other oxidizing agents—undesirable secondary effects of acoustic cavitation—which subsequently attack the target molecules [15].

To evaluate the effect of extraction time on the extraction process, different times were tested from 5 to 20 min, maintaining the other parameters constant: ultrasound amplitude 30%, ethanol concentration 60% (*v*/*v*), extraction temperature 50 °C, and S/P ratio 20:1 (mL/g).

As illustrated in Table 6 the maximum TPC and TFC values were recorded at 10 min, these values being significantly higher than those observed at 5 or 15 min, thereby suggesting that a 5 min extraction time is insufficient for complete extraction, whereas a 15 min duration induces the aforementioned degradation phenomena. Furthermore, antioxidant activity and DPPH radical inhibition followed a similar trend, showing significantly higher efficacy at 10 min. Quantitative HPLC analysis of quercetin content (Table 6) further corroborated these findings, indicating that the highest concentration of quercetin was recovered at 10 min. Consequently, the integration of these analytical results identifies 10 min as the optimal duration for maximizing the extraction yield of antioxidant compounds from onion skins. Accordingly, a range of 5–15 min was selected as the working interval for subsequent BBD optimization.

### 3.2. Energy Considerations

The ultrasound power was recorded during all extractions and a wattmeter was used for the conventional extraction to measure the power input for the heating plate. The total and specific energies for each extraction are presented in Table 7.

The total energy introduced into the systems was determined using these recorded powers and, further, the specific energy was calculated using the following equation:

(2) $$E_{s}=E_{total}/m_{Bioactive\;Compounds}\;\left[kJ/g\;of\;Bioactive\;Compounds\right]$$

where *E_s_* is the specific energy [kJ/g of bioactive compounds], *E_total_* is the total energy introduced into the system [kJ], and *m_Bioactive Compounds_* is the total amount of phytocompounds (sum of polyphenols and flavonoids) obtained by each extraction method [g].

The energy consumption for conventional extraction (378 kJ) is considerably higher than that of UAE, which ranged from 5.23 to 21.65 kJ. The acoustic energy delivered by the ultrasonic processor constitutes a critical operating parameter in the extraction of bioactive compounds.

Total energy consumption increased with increasing extraction time and ultrasound amplitude and decreased with an increase in ethanol in the extraction solvent. Sonication significantly improves extraction efficiency by accelerating mass transfer, which in turn lowers the unit energy demand in the early phase of the process. The observed increase in energy consumption with extraction time indicates that sonication did not induce changes in solvent properties that could alter energy transmission, such as viscosity or acoustic impedance. Under these conditions, the amplitude of ultrasonic vibrations exerted a stronger influence on extraction performance than treatment duration. Notably, an equivalent percentage increase in vibration amplitude, compared with extraction time, resulted in a substantially greater transfer of energy to the extraction medium [42]. Regarding the ethanol concentration in water, it can be noticed in Table 7 that the lowest specific energy is achieved for 60% ethanol in the extraction solvent. This can be explained by the fact that ultrasonic processors consume more energy in liquids with higher acoustic impedance; consequently, the energy generated during extraction in water is greater than that observed when ethanol is used as the solvent [43].

The energy analysis summarized in Table 7 indicates that UAE represents the most sustainable extraction approach, exhibiting the lowest specific energy consumption across the investigated extraction conditions considering the antioxidant compounds (polyphenols and flavonoids) within the same extract. Moreover, the non-conventional extraction technique is associated with substantially lower energy requirements than conventional heating extraction, regardless of the operating conditions applied. The energy consumption obtained in the present study agrees with previous reports on UAE from onion by-products. Benito-Román et al. [37] reported that UAE of flavonoids from dry onion skin waste reduced the extraction time to less than 5 min and the energy input to below 10 kJ/g dry onion skin waste. Moreover, they observed that energy inputs above 10 kJ/g did not further improve the extraction yield, suggesting that excessive sonication may not be beneficial from an energetic point of view. Therefore, the short extraction time used in the present study may be advantageous for reducing energy demand while maintaining efficient recovery of bioactive compounds.

Therefore, to produce extracts enriched in bioactive compounds with pronounced antioxidant potential, UAE may be considered the most advantageous method from both energetic and functional perspectives. From a scalability perspective, UAE presents potential for industrial application due to its reduced extraction time, moderate operating conditions, and lower solvent requirements. Nevertheless, further scale-up should consider ultrasound power density, reactor geometry, mixing efficiency, and temperature control to ensure uniform cavitation and reproducible extraction efficiency at larger processing volumes.

### 3.3. Box–Behnken Design (BBD)

#### 3.3.1. Mathematical Model Selection and Fitting

Preliminary one-factor-at-a-time (OFAT) experiments were conducted to identify the most influential UAE variables and to define suitable operating ranges. In the present study, six variables were evaluated regarding their influence on TPC, TFC, quercetin content, CUPRAC antioxidant activity, and DPPH RSA. Using ANOVA, the extraction parameters with statistically significant influence were determined. Ethanol concentration was the most significant factor, followed by ultrasound amplitude and extraction time. The preliminary parametric study showed that ethanol concentration in combination with sonication power gave maximum bioactive content and antioxidant activity of extracts from yellow onion skins. According to the OFAT study the significant parameters were identified and three factors were selected for further optimization: ethanol concentration (X_1_), ultrasound amplitude (X_2_), and extraction time (X_3_). A BBD design with 17 experiments was studied. In a factorial design these three parameters at three levels should be investigated by 3^3^ (27) experiments. However, this number of experiments was reduced to 17 using a Box–Behnken experimental design. The experimental design matrix containing independent factors, experimental results and predicted responses for yellow onion peel extracts is presented in Table S1.

The bioactive compounds ranged from 78.16 to 97.16 mg GAE/g DM for TPC, from 24.77 to 27.46 mg QE/g DM for TFC, and from 4.74 to 6.07 mg Q/gDM for quercetin, while CUPRAC antioxidant activity values ranged from 141.22 to 161.87 mg TE/g DM and those of the DPPH RSA from 59.03 to 75.98%.

The goal was to maximize the multi-response parameters: TPC (Y_1_), TFC (Y_2_), quercetin content (Y_3_), CUPRAC antioxidant activity (Y_4_), and DPPH RSA (Y_5_), while keeping the independent parameters (X_1_ to X_3_) within their established low and high limits (Table 1). Multiple regression analysis was used to fit the experimental data and generate predictive equations for multi-response parameters: TPC, TFC, quercetin content, CUPRAC antioxidant activity, and DPPH RSA, expressed as functions of the independent variables within the studied range.

The model coefficients were predicted to evaluate the influence between variables and responses. The fitted surface models for response parameters of UAE of yellow onion peels are expressed in Equations (3)–(7).

(3) $$\mathrm{TPC}\;(\mathrm{mgGAE}/\mathrm{gDM})=92.82-4.86X_{1}+0.3675X_{2}+3.04X_{3}-0.5775X_{1}X_{2}-3.80X_{1}X_{3}-3.09X_{2}X_{3}-7.57X_{1}^{2}-1.51X_{2}^{2}-0.5045X_{3}^{2}$$

(4) $$\mathrm{TFC}\;(\mathrm{mgQE}/\mathrm{gDM})=27.06+0.1538X_{1}+0.2875X_{2}+0.2412X_{3}+0.5275X_{1}X_{2}+0.32X_{1}X_{3}-0.9411X_{1}^{2}-0.5236X_{2}^{2}$$

(5) $$\mathrm{QC}\;(\mathrm{mgQ}/\mathrm{gDM})=5.83-0.4464X_{1}-0.0124X_{2}-0.0789X_{3}-0.0878X_{1}X_{3}-0.0130X_{2}X_{3}-0.3256X_{1}^{2}-0.0930X_{2}^{2}-0.0617X_{3}^{2}$$

(6) $$\mathrm{CUPRAC}\;(\mathrm{mgTE}/\mathrm{gDM})=157.37-4.05X_{1}+0.1852X_{2}-0.3697X_{3}-5.06X_{1}X_{2}-1.41X_{1}X_{3}-2.50X_{2}X_{3}-6.25X_{1}^{2}-4.01X_{3}^{2}$$

(7) $$\mathrm{DPPH}\;\mathrm{RSA}\;(\%)=73.07-3.89X_{1}+0.4717X_{2}+2.42X_{3}+0.0965X_{1}X_{2}-4.39X_{1}X_{3}+1.27X_{2}X_{3}-8.73X_{1}^{2}$$

The model equation contains only significant terms (i.e., *p*-value < 0.05) except those necessary to support the hierarchical model. For each response, an initial quadratic model was fitted. Non-significant terms were removed by backward elimination to obtain a parsimonious model, while maintaining model hierarchy. Final models were selected based on ANOVA significance (*p* < 0.05), non-significant lack of fit (*p* > 0.05), adequate precision, and agreement between adjusted and predicted coefficients of determination (Adj-R^2^ − Pred-R^2^ < 0.2). The outcomes of the regression analysis, including model fit and adequacy indicators, are summarized in Table S2. All five models had *p*-values < 0.05, lack of fit is not significant with *p*-values higher than 0.05, and F-values of 5.50 (TPC), 9.26 (TFC), 11.44 (quercetin), 6.42 (CUPRAC AA), and 29.85 (DPPH RSA), demonstrating that the lack of fit is not significant relative to the pure error. For all responses, the predicted R^2^ values (0.8479 for TPC, 0.8781 for TFC, 0.9196 for quercetin, 0.8652 for AA, and 0.9587 for RSA) agree with the adjusted R^2^ values (0.6523 for TPC, 0.7833 for TFC, 0.8392 for quercetin, 0.7305 for AA, and 0.9266 for RSA), the differences being less than 0.2. These results indicated the good fitting accuracy of all five models, and the possibility to be used for the subsequent prediction and correlation analysis.

#### 3.3.2. Effect of UAE on Bioactive Compound Content of Yellow Onion Peel Extracts

**Figure 1:**
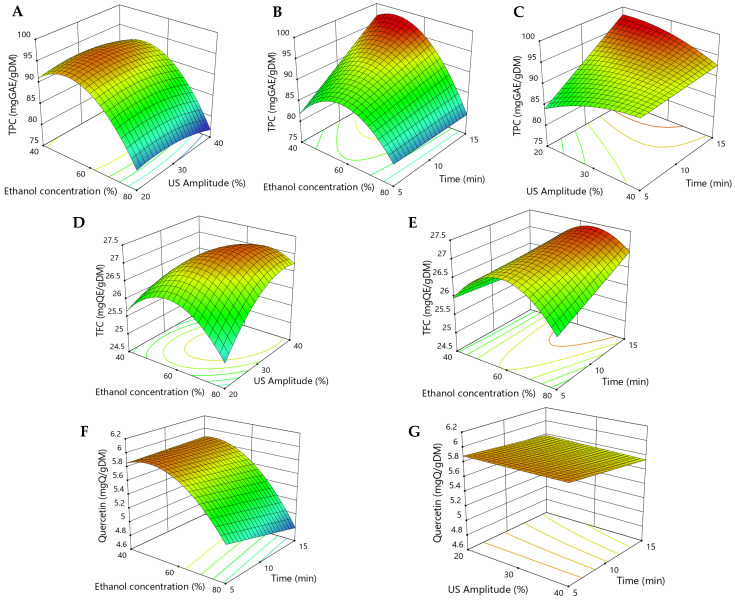
Response surface 3D plots showing the effects of ethanol concentration, US amplitude, and time on the polyphenol content (**A**–**C**), flavonoid content (**D**,**E**) and quercetin content (**F**,**G**) from yellow onion peel extracts.

In order to determine the effect of the significant factors and to demonstrate the interactions among the independent variables in the extraction of bioactive compounds from yellow onion peels, 3D response surface plots were generated (Figure 1A–G).

The effect of ethanol concentration was evaluated in the range of 40–80% on the bioactive compound extraction efficiency. Figure 1A,B,D–F present 3D plots illustrating the effect of solvent concentration on the onion peel extract. The interaction of ethanol concentration with US amplitude (extraction time constant at 10 min, X_3_ = 0, Figure 1A,D) and extraction time (US amplitude constant at 30%, X_2_ = 0, Figure 1B,E,F) had a greater effect on the TPC, TFC, and quercetin content. For all responses ethanol concentration (X_1_) and the quadratic term $X_{1}^{2}$ were significant (*p* < 0.05), indicating curvature. As shown in the plots, increasing ethanol concentration from 40% to 60% increased the TPC, TFC and quercetin content and reached the maximum at 50% ethanol in water for TPC and quercetin content and 60% for TFC. On the other hand, an additional increase in solvent concentration to 80% reduced the bioactive compound extraction (Figure 1A,B,D–F). At low concentrations, ethanol can improve cell permeability and extraction of polyphenolic compounds and flavonoids. Conversely, high ethanol concentrations can cause protein denaturation and cell dehydration, which impede the extraction process and reduce the bioactive compound content [35]. These results were in agreement with other literature studies on onion extracts that reported the same conclusions about the effect of ethanol concentration on polyphenol and flavonoid recovery from onion peels [37,44,45].

The effect of ultrasound amplitude on the extraction process is illustrated in Figure 1C,D,G. These three-dimensional surface plots depict the response of TPC, TFC, and quercetin yield, respectively, as functions of amplitude percentage and extraction time, while the ethanol concentration was maintained at a constant 60% (coded as X_1_ = 0, Figure 1C,G). The highest TPC was obtained at an US amplitude between 20 and 30% and decreased with increasing ultrasound power. However, at the highest amplitude percentage values, the value of the TFC response decreased at extraction times longer than 14 min, as can be observed in Figure 1C. The decline in bioactive compound content at high US amplitude can be explained by the violent collapse of cavitation bubbles when ultrasonic power increased. The size of resonating cavitation bubbles is directly influenced by the power of the applied ultrasound. As bubble diameter increases, the intensity of their collapse also rises, leading to more forceful implosions. These events cause mechanical disruption of the plant tissue, including fragmentation and the formation of pores. Such structural changes enhance mass transfer by improving diffusivity within the matrix, ultimately resulting in higher extraction yields. The high US amplitude could also degrade the bioactive compounds, thereby reducing their extractability [15].

The effect of extraction time on the recovery of phenolic compounds is illustrated in Figure 1B,C and for recovery of flavonoids in Figure 1E. For TFC, extraction time (X_3_) and the quadratic term $X_{3}^{2}$ were significant (*p* < 0.05), indicating curvature. TFC showed a steady increase between 10 and 15 min, peaking at around 13 min. The ultrasonication time also affected the quercetin content (Figure 1G). Numerous studies have reported that increasing the duration of ultrasound-assisted extraction generally improves the yield of bioactive compounds, particularly during the initial stages of the process, where a near-linear increase is often observed. This trend is largely attributed to the mechanical disruption of plant cell walls by ultrasonic waves, which enhances the release of intracellular compounds into the surrounding solvent. Moreover, prolonged extraction may facilitate oxidative transformations, especially in phenolic compounds, which are prone to oxidation in the presence of oxygen. Such oxidative reactions can alter their chemical structure, thereby diminishing their biological efficacy and overall extract quality. Extended extraction periods may also co-extract undesirable components or increase the concentration of interfering substances in the final extract [15].

#### 3.3.3. Effect of UAE on Antioxidant Capacity of Yellow Onion Peel Extracts

**Figure 2:**
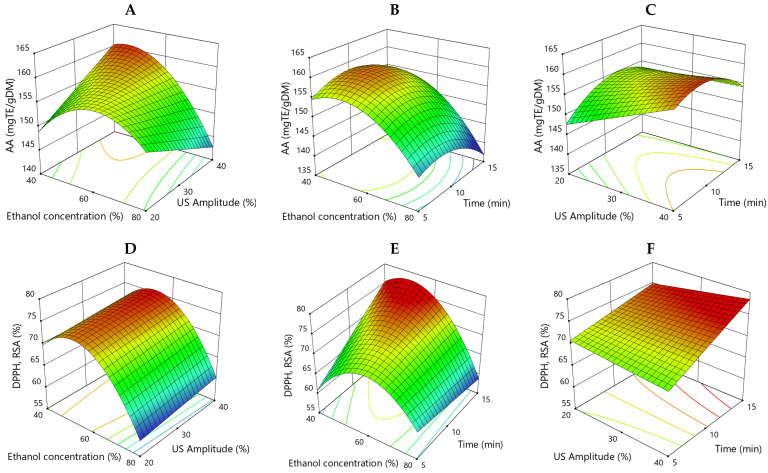
Response surface 3D plots showing the effects of ethanol concentration, US amplitude, and time on the antioxidant capacity of yellow onion peel extracts expressed as CUPRAC AA (**A**–**C**) and DPPH RSA (**D**–**F**).

The beneficial features of polyphenols were attributed to their antioxidant capacity. Therefore, these antioxidants may act as free radical scavengers, potential chelators of metal ions, and reducing agents and reduce oxidative stress. In this study two techniques were used to analyze the antioxidant activity of onion peel extracts: CUPRAC antioxidant activity (CUPRAC AA) and DPPH RSA. The effects of ethanol concentration, US amplitude, and extraction time and their interactions on CUPRAC AA and DPPH RSA of yellow onion skin extracts are shown in 3D response surface plots in Figure 2.

Figure 2A,D show the interactive effect of ethanol concentration and US amplitude on the CUPRAC AA and DPPH RSA respectively, at a fixed extraction time (X_3_ = 0). The AA was highest at an ethanol concentration around 50% and an US amplitude of 35–40%. The DPPH RSA was high at a 50–60% ethanol concentration with no significant effect of the sonication power. The increase in solvent concentration from 40% to 60% increased the antioxidant capacity (AA and RSA), then the two responses dropped significantly with an increase in the solvent concentration from 60% to 80%. Figure 2B,E present the response surface plots for the interactive effect of ethanol concentration and extraction time on the two response values at a fixed US amplitude (X_2_ = 0). For both factors, ethanol concentration (X_1_) and extraction time (X_3_) and the quadratic terms $X_{1}^{2}$ and $X_{3}^{2}$ were significant (*p* < 0.05) indicating curvature. An increase in solvent concentration from 40% to 60% improved the antioxidant activities. In the case of CUPRAC AA a peak value is observed at 50% ethanol concentration and 12 min extraction time, while DPPH RSA increased significantly with an increase in extraction time from 10 to 15 min. In Figure 2C,F the 3D plots for the interactive effect of the US amplitude and extraction time on the antioxidant capacities at a fixed ethanol concentration (X_1_ = 0) are shown. The highest values of CUPRAC AA and DPPH RSA were reached at the highest US amplitude. These results also correlated with bioactive compound contents, TPC and TFC measured in the yellow onion peel extract, suggesting that these compounds contribute to the observed antioxidant activity. Based on these findings, it can be inferred that the optimized extract demonstrates comparable properties to those reported in previous studies.

#### 3.3.4. Model Validation and Optimization

Multi-response optimization was performed using the desirability function in Design-Expert v11 to simultaneously maximize TPC, TFC, quercetin content, CUPRAC antioxidant activity, and DPPH RSA. The extraction factors – ethanol concentration (X_1_), ultrasound amplitude (X_2_), and extraction time (X_3_)—were constrained within their studied ranges. The optimal operating conditions were selected based on the maximum overall desirability (D), and the corresponding predicted values for all responses were obtained from the final fitted models. To validate the multi-response optimum, confirmatory experiments were conducted in the predicted optimal conditions. Because the numerical solution can include non-integer factor levels, the optimal settings were rounded to experimentally practical values and the responses were measured in triplicate (mean ± SD). The experimental values were compared with model predictions and the prediction error was calculated using Equation (7) [18]:

(8) $$\%\;\mathrm{Error}\;=\;\frac{\left|\mathrm{Experimental}\;\mathrm{values}\;-\mathrm{Predicted}\;\mathrm{Values}\right|}{\mathrm{Predicted}\;\mathrm{values}\;}\;\times \;100$$

The response surface methodology (RSM) predicted the optimal extraction conditions as follows: optimal ethanol concentration of 53%, optimal US amplitude of 30%, and optimal extraction time of 13 min for maximization of all factors based on the maximum overall desirability (D = 0.868). The predicted values and the experimental results are presented in Table 8.

Confirmatory experiments performed in optimum conditions showed good agreement with predicted values, with prediction errors of 0.50–3.22% across responses, confirming the adequacy of the models and robustness of the desirability-based optimum. The close correspondence between the predicted and observed values confirmed the applicability of the developed models for the optimization of bioactive compound extraction and the antioxidant capacity of yellow onion peel extracts. Furthermore, response surface methodology (RSM) proved to be an effective tool for maximizing the extraction of bioactive antioxidant compounds from yellow onion peel.

## 4. Conclusions

Yellow onion peels are generated in large quantities as an agro-industrial by-product and, as this study confirms, represent a genuinely rich source of quercetin and other antioxidant phytochemicals that deserve more attention than they currently receive. This study investigated the influence of extraction parameters on the ultrasound-assisted extraction of bioactive compounds from yellow onion peels using both the one-factor-at-a-time (OFAT) approach and the Box–Behnken design (BBD) for process optimization. Initially, six quantitative variables were evaluated through the OFAT design: ultrasound power (0–40 W/g DM), solvent concentration (0–100%), plant-to solvent ratio (10–30 mL/g), temperature (40–70 °C), and extraction time (5–20 min). Statistical screening revealed that the ethanol concentration, ultrasound power and extraction time significantly influenced the main response variables—total phenolic content (TPC), total flavonoid content (TFC), quercetin content and antioxidant capacity (CUPRAC and DPPH). These key factors were subsequently optimized using the BBD. Under the identified optimal conditions (ethanol concentration = 53%, US amplitude = 30%, and extraction time = 13 min), and maximum overall desirability (D = 0.868), the experimentally determined response values closely matched those predicted by the models, confirming their adequacy and predictive capability.

The novelty of this study resides in the integration of optimized ultrasound-assisted extraction parameters with the valorization of onion peel waste as a sustainable and efficient source of quercetin, supporting the development of functional ingredients and promoting circular economy approaches in agro-industrial processing. The optimization strategy developed in this work can be extended to different extraction conditions or additional process variables. As demonstrated by the energy assessment UAE can be regarded as the greenest extraction technique, as it resulted in the lowest specific energy consumption under the evaluated conditions when considering the simultaneous recovery of antioxidant bioactive compounds. Extracts produced under optimized parameters, characterized by elevated levels of phenolic acids and flavonoids, represent promising antioxidant-rich ingredients for applications in functional foods, cosmetics, and food supplements. Future studies should evaluate the scalability of the process under pilot-scale conditions, including energy efficiency, economic feasibility, reproducibility, and process control at larger volumes. In addition, further research should investigate the stability of the obtained extracts during storage and their potential incorporation into food, pharmaceutical, or nutraceutical formulations.

## Figures and Tables

**Table 1:** The range of independent factors in the UAE of yellow onion peels.

Independent Factors	Unit	Lower Limit (−1)	Center Point (0)	Upper Limit (+1)
Ethanol concentration (X_1_)	%	40	60	80
Ultrasound amplitude (X_2_)	%	20	30	40
Extraction time (X_3_)	min	5	10	15

**Table 2:** Effect of ultrasonic power on bioactive compound content and antioxidant activity of yellow onion extracts. The significant differences among groups (*p* < 0.05, ANOVA) are indicated by different letters (a–e). Results are expressed as the mean of triplicates ± SD.

US Specific Power	Duty Cycle	US Amplitude	Bioactive Content			Antioxidant Activity		
			TPC	TFC	Quercetin	CUPRAC	DPPH	
							RSA	IC50
W/mL	%	%	mg GAE/g DM	mg QE/g DM	mg/g DM	mg TE/g DM	%	mg/L
0	0	0	81.03 ± 1.03 ^d^	24.24 ± 1.50 ^e^	5.51 ± 0.23 ^c^	147.11 ± 2.71 ^c^	38.64 ± 3.11 ^d^	742.01 ± 29.80 ^a^
0.188	50	20	86.19 ± 1.70 ^c^	27.03 ± 1.60 ^d^	6.26 ± 0.12 ^a,b^	157.96 ± 4.10 ^b^	51.99 ± 0.75 ^c^	720.81 ± 56.51 ^a,b^
0.436	100	20	94.03 ± 3.90 ^a,b^	29.32 ± 1.65 ^c^	6.12 ± 0.43 ^b^	167.76 ± 3.16 ^a^	56.23 ± 1.47 ^b^	677.90 ± 51.07 ^a,b^
0.908	100	30	96.04 ± 2.86 ^a^	38.08 ± 1.59 ^a^	6.30 ± 0.75 ^a^	171.87 ± 4.05 ^a^	67.84 ± 0.83 ^a^	576.76 ± 3.27 ^c^
1.338	100	40	92.16 ± 1.63 ^a,b^	32.18 ± 4.44 ^b,c^	6.30 ± 0.19 ^a^	160.09 ± 3.02 ^b^	56.89 ± 2.84 ^b^	656.60 ± 41.32 ^b^
1.779	100	60	89.94 ± 1.06 ^b,c^	27.77 ± 0.34 ^c,d^	5.64 ± 0.37 ^c^	158.70 ± 3.77 ^b^	56.37 ± 1.19 ^b^	655.32 ± 52.03 ^b^

**Table 3:** Effect of ethanol concentration on bioactive compound content and antioxidant activity of yellow onion extracts. The different letters (a–f) highlight the significant differences between groups (*p* < 0.05, ANOVA). Results are expressed as the mean of triplicates ± SD.

Ethanol Concentration	Bioactive Content			Antioxidant Activity		
	TPC	TFC	Quercetin	CUPRAC	DPPH	
					RSA	IC50
%	mg GAE/g DM	mg QE/g DM	mg/g DM	mg TE/g DM	%	mg/L
0	37.38 ± 1.97 ^d^	13.49 ± 0.45 ^f^	2.29 ± 0.05 ^e^	75.89 ± 1.94 ^d^	33.21 ± 4.45 ^d^	987.81 ± 10.23 ^a^
20	74.86 ± 5.00 ^c^	20.74 ± 0.64 ^d^	4.87 ± 0.19 ^c^	126.57 ± 3.21 ^c^	56.14 ± 1.65 ^b^	585.73 ± 11.99 ^c^
40	92.70 ± 0.27 ^a^	25.60 ± 0.43 ^c^	5.89 ± 0.08 ^b^	153.93 ± 2.90 ^b^	64.92 ± 1.63 ^a^	582.15 ± 16.33 ^c^
60	96.04 ± 2.86 ^a^	38.08 ± 1.59 ^a^	6.30 ± 0.15 ^a^	171.87 ± 4.05 ^a^	67.84 ± 0.83 ^a^	576.76 ± 3.27 ^c^
80	84.04 ± 2.70 ^b^	27.83 ± 1.28 ^b^	5.66 ± 0.11 ^b^	134.55 ± 0.44 ^c^	38.73 ± 0.41 ^c^	625.03 ± 19.34 ^b^
100	27.71 ± 0.33 ^e^	15.68 ± 0.45 ^e^	3.29 ± 0.21 ^d^	57.72 ± 0.44 ^e^	26.05 ± 2.06 ^e^	628.82 ± 36.60 ^b^

**Table 4:** Effect of solvent-to-plant ratio on bioactive compound content and antioxidant activity of yellow onion extracts. The significant differences between groups (*p* < 0.05, ANOVA) are indicated by different letters (a–c). Results are expressed as the mean of triplicates ± SD.

S/P Ratio	Bioactive Content			Antioxidant Activity		
	TPC	TFC	Quercetin	CUPRAC	DPPH	
					RSA	IC50
mL/g	mg GAE/g DM	mg QE/g DM	mg/g DM	mg TE/g DM	%	mg/L
10	79.19 ± 3.80 ^b^	24.27 ± 1.85 ^c^	6.10 ± 0.07 ^b^	104.53 ± 4.14 ^b^	78.49 ± 2.66 ^a^	592.66 ± 35.05 ^b^
20	96.04 ± 2.86 ^a^	38.08 ± 1.59 ^a^	6.30 ± 0.15 ^a^	171.87 ± 4.05 ^a^	67.84 ± 0.83 ^b^	576.76 ± 3.27 ^c^
30	75.50 ± 2.47 ^b^	28.18 ± 0.88 ^b^	6.24 ± 0.24 ^a^	166.50 ± 5.24 ^a^	68.46 ± 1.31 ^b^	639.81 ± 34.85 ^a^

**Table 5:** Effect of temperature on bioactive compound content and antioxidant activity of yellow onion extracts. The significant differences amongst groups (*p* < 0.05, ANOVA) are emphasized by different letters (a–d). Results are expressed as the mean of triplicates ± SD.

Temperature	Bioactive Content			Antioxidant Activity		
	TPC	TFC	Quercetin	CUPRAC	DPPH	
					RSA	IC50
°C	mg GAE/g DM	mg QE/g DM	mg/g DM	mg TE/g DM	%	mg/L
40	84.19 ± 4.44 ^b^	33.84 ± 1.02 ^c^	5.91 ± 0.05 ^b^	171.87 ± 3.50 ^a^	45.11 ± 2.13 ^b^	648.21 ± 24.15 ^c^
50	96.04 ± 2.86 ^a^	38.08 ± 1.59 ^b^	6.30 ± 0.15 ^a^	141.90 ± 4.05 ^c^	67.84 ± 0.83 ^a^	576.76 ± 3.27 ^b^
60	89.22 ± 2.88 ^a,b^	41.62 ± 1.06 ^a^	6.13 ± 0.11 ^a^	163.17 ± 4.94 ^b^	39.27 ± 2.84 ^c^	602.82 ± 11.66 ^b^
70	85.56 ± 6.13 ^b^	40.75 ± 4.11 ^a^	5.88 ± 0.32 ^b^	1431.18 ± 3.18 ^c^	32.87 ± 3.63 ^d^	634.51 ± 20.72 ^c^

**Table 6:** Effect of extraction time on bioactive compound content and antioxidant activity of yellow onion extracts. The significant differences between groups (*p* < 0.05, ANOVA) are highlighted by different letters (a–d). Results are expressed as the mean of triplicates ± SD.

Time	Bioactive Content			Antioxidant Activity		
	TPC	TFC	Quercetin	CUPRAC	DPPH	
					RSA	IC50
min	mg GAE/g DM	mg QE/g DM	mg/g DM	mg TE/g DM	%	mg/L
5	88.98 ± 3.55 ^c^	35.03 ± 2.67 ^b^	5.71 ± 0.11 ^b^	150.04 ± 1.90 ^c^	38.41 ± 2.91 ^c^	611.82 ± 11.29 ^a^
10	96.04 ± 2.86 ^a^	38.08 ± 1.59 ^a^	6.30 ± 0.15 ^a^	171.87 ± 4.05 ^a^	67.84 ± 0.83 ^a^	576.76 ± 3.27 ^c^
15	93.25 ± 3.10 ^b^	36.76 ± 1.08 ^b^	5.72 ± 0.09 ^b^	158.51 ± 1.61 ^b^	43.17 ± 1.42 ^b^	592.95 ± 29.01 ^b^
20	91.02 ± 1.85 ^b,c^	36.14 ± 2.53 ^b^	5.45 ± 0.24 ^c^	144.22 ± 3.75 ^d^	40.73 ± 1.98 ^c^	582.15 ± 17.31 ^b^

**Table 7:** Energy consumption during UAE of antioxidant phytocompounds from yellow onion peels.

Method Description	Total Energy (kJ)	Total Bioactive Compounds (g/g DM)	Specific Energy (kJ/g of Bioactive Compounds)
US Amplitude 20% (5 on/5 off), 50 °C, 60% Ethanol, 10 min, S/P Ratio 20/1	2.25	0.113	19.91
US Amplitude 20%, 50 °C, 60% Ethanol, 10 min, S/P Ratio 20/1	5.23	0.123	42.42
US Amplitude 30%, 50 °C, 60% Ethanol, 10 min, S/P Ratio 20/1	10.89	0.134	81.20
US Amplitude 40%, 50 °C, 60% Ethanol, 10 min, S/P Ratio 20/1	16.06	0.124	129.14
US Amplitude 60%, 50 °C, 60% Ethanol, 10 min, S/P Ratio 20/1	21.65	0.118	183.96
US Amplitude 30%, 50 °C, 0% Ethanol, 10 min, S/P Ratio 20/1	13.88	0.051	272.833
US Amplitude 30%, 50 °C, 20% Ethanol, 10 min, S/P Ratio 20/1	12.32	0.096	128.881
US Amplitude 30%, 50 °C, 40% Ethanol, 10 min, S/P Ratio 20/1	11.41	0.118	96.416
US Amplitude 30%, 50 °C, 80% Ethanol, 10 min, S/P Ratio 20/1	10.38	0.112	92.777
US Amplitude 30%, 50 °C, 100% Ethanol, 10 min, S/P Ratio 20/1	9.65	0.043	222.494
US Amplitude 30%, 50 °C, 60% Ethanol, 10 min, S/P Ratio 10/1	9.17	0.103	88.633
US Amplitude 30%, 50 °C, 60% Ethanol, 10 min, S/P Ratio 30/1	11.85	0.104	114.246
US Amplitude 30%, 40 °C, 60% Ethanol, 10 min, S/P Ratio 20/1	13.38	0.118	113.353
US Amplitude 30%, 60 °C, 60% Ethanol, 10 min, S/P Ratio 20/1	8.76	0.131	66.929
US Amplitude 30%, 70 °C, 60% Ethanol, 10 min, S/P Ratio 20/1	6.38	0.126	50.479
US Amplitude 30%, 50 °C, 60% Ethanol, 5 min, S/P Ratio 20/1	5.81	0.124	46.819
US Amplitude 30%, 50 °C, 60% Ethanol, 15 min, S/P Ratio 20/1	16.35	0.130	125.744
US Amplitude 30%, 50 °C, 60% Ethanol, 20 min, S/P Ratio 20/1	21.53	0.127	169.306
Conventional, 50 °C, 60% Ethanol, 10 min, S/P Ratio 20/1	378	0.105	3609.77

**Table 8:** Comparison between predicted and experimentally obtained values of the extraction response variables under the optimized processing conditions for yellow onion peel extracts (optimal extraction conditions: ethanol concentration = 53%, US amplitude = 30%, and extraction time = 13 min and desirability (D = 0.868).

Response Variable (Unit)	Optimal Predicted Values	Optimal Experimental Values	Prediction Error, %
TPC (mg GAE/gDM)	96.15	904 ± 2.07	1.75
TFC (mg QE/gDM)	26.99	27.76 ± 2.76	2.84
Quercetin content (mg Q/gDM)	5.88	6.03 ± 0.29	3.22
CUPRAC AA (mg TE/gDM)	156.41	161.44 ± 4.66	0.50
DPPH RSA (%)	75.98	76.36 ± 3.58	2.42

## Data Availability

The original contributions presented in the study are included in the article and in the Supplementary Materials.

## Supplementary Materials

The following supporting information can be downloaded at: https://www.mdpi.com/article/10.3390/antiox15050632/s1, Table S1: Experimental design conditions with independent factors, experimental results and predicted responses for yellow onion peel extracts; Table S2: The model selection for each response of the yellow onion peel extract. Figure S1: HPLC chromatogram for standard quercetin; Figure S2: HPLC chromatogram of extracts obtained from yellow onion peels under optimum extraction conditions.
