# Supplementary material for: Sustainable Extraction of Antioxidant Phytocompounds from Yellow Onion Wastes for Value-Added Product Development
Source: Antioxidants (Basel). 2026 May 15;15(5):632. doi: 10.3390/antiox15050632 (PMC13203277; doi:10.3390/antiox15050632)
Supplement: Supplementary file 1 [file antioxidants-15-00632-s001.zip › antioxidants-4271506-supplementary.pdf]

Sustainable Extraction of Antioxidant Phytocompounds from Yellow Onion Wastes for Value-Added Product Development

Anca M. Rosca [1], Adina I. Gavrila [1,*], Ioan Calinescu [1], Christina Zalaru [2], Mihaela D. Popescu, Alexandra Ene-Manea [3,4], and Justinian A. Tomescu [1,3*]

[1] Faculty of Chemical Engineering and Biotechnologies, National University of Science and Technology Politehnica Bucharest, 011061 Bucharest, Romania; anca_maria.rosca@stud.fim.upb.ro (A.M.R.), ioan.calinescu@upb.ro (I.C.), mihadana2001@gmail.com (M.D.P.)
[2] Faculty of Chemistry, University of Bucharest, 050663 Bucharest, Romania, chmzalaru@gmail.com (C.Z.)
[3] HOFIGAL Export-Import S.A, 042124 Bucharest, Romania;
[4] Faculty of Agriculture, Department of Biology, University of Agronomic Sciences and Veterinary Medicine of Bucharest, 011464 Bucharest, Romania; manea_alexa96@yahoo.com (A.E.M.)
* Correspondence: adina.gavrila@upb.ro (A.I.G.); tomescu.justinian@gmail.com (J.A.T)

Table S1. Experimental design conditions with independent factors, experimental results and predicted responses for yellow onion peels extracts.

Runs	X_1	X_2	X_3	Experimental values					Predicted values				
				Y_1	Y_2	Y_3	Y_4	Y_5	Y_1	Y_2	Y_3	Y_4	Y_5
1	60 (0)	30 (0)	10 (0)	96.04	27.08	5.90	161.87	74.00	92.82	27.06	5.83	157.84	73.07
2	80 (+1)	30 (0)	5 (-1)	79.92	25.79	5.07	145.24	61.42	80.64	25.71	5.16	144.03	62.42
3	60 (0)	30 (0)	10 (0)	86.79	26.42	5.71	152.73	72.25	92.82	27.06	5.83	157.84	73.07
4	40 (-1)	30 (0)	5 (-1)	81.22	26.24	5.96	148.94	59.03	82.78	26.05	5.88	152.12	61.41
5	40 (-1)	40 (+1)	10 (0)	89.88	25.29	5.76	160.99	69.18	88.97	25.20	5.84	159.81	68.70
6	40 (-1)	20 (-1)	10 (0)	88.17	25.40	5.78	149.72	69.39	88.24	25.68	5.87	149.33	67.75
7	60 (0)	30 (0)	10 (0)	96.30	27.33	5.85	154.78	72.87	92.82	27.06	5.83	157.84	73.07
8	60 (0)	40 (+1)	5 (-1)	92.46	26.43	5.77	156.40	71.22	91.23	26.58	5.74	155.82	69.85
9	80 (+1)	40 (+1)	10 (0)	78.16	26.77	5.01	141.22	60.22	79.25	26.57	4.95	141.61	60.92
10	60 (0)	30 (0)	10 (0)	87.98	27.46	5.60	159.59	73.58	92.82	27.06	5.83	157.84	73.07
11	60 (0)	30 (0)	10 (0)	97.01	26.86	6.07	160.25	74.35	92.82	27.06	5.83	157.84	73.07
12	60 (0)	40 (+1)	15 (+1)	90.08	26.93	5.57	148.70	75.98	91.13	27.07	5.58	150.08	77.24
13	60 (0)	20 (-1)	5 (-1)	85.37	26.05	5.74	151.83	71.87	84.32	26.01	5.76	150.45	71.45
14	80 (+1)	30 (0)	15 (+1)	80.68	26.72	4.74	143.65	60.12	79.12	26.84	4.83	143.29	58.49
15	60 (0)	20 (-1)	15 (+1)	95.34	26.90	5.60	154.12	71.53	96.57	26.49	5.61	154.71	73.75
16	40 (-1)	30 (0)	15 (+1)	97.16	25.89	5.99	152.98	75.29	96.44	25.89	5.90	151.38	75.03
17	80 (+1)	20 (-1)	10 (0)	78.76	24.77	5.09	150.17	60.05	78.51	24.94	4.97	151.35	59.98

X_1: Ethanol concentration (%), X_2: US Amplitude (%), and X_3: Extraction time (min). Y_1: TPC (mgGAE/gDM), Y_2: TFC (mgQE/gDM), Y_3: Quercetin content (mgQ/gDM), Y_4: CUPRAC antioxidant activity (mg TE/gDM), and Y_5: DPPH RSA (%).

Table S2. The model selection for each response of the yellow onion peels extract.

Parameters	Y_1	Y_2	Y_3	Y_4	Y_5
**Mathematical model**	**Quadratic**	**Reduced Quadratic**	**Reduced Quadratic**	**Reduced Quadratic**	**Reduced Quadratic**
Model significance	0.0133	0.0017	0.0012	0.0082	< 0.0001
Model F-value	5.50	9.26	11.44	6.42	29.85
Lack of fit *p*-value	0.9624	0.7629	0.7529	0.8532	0.0532
Lack of fit F-value	0.1320	0.5069	0.4796	0.3184	6.03
R^2	0.8479	0.8781	0.9196	0.8652	0.9587
Adjusted-R^2	0.6523	0.7833	0.8392	0.7305	0.9266
Adequate precision	5.8435	8.8528	9.4594	8.3169	16.4641

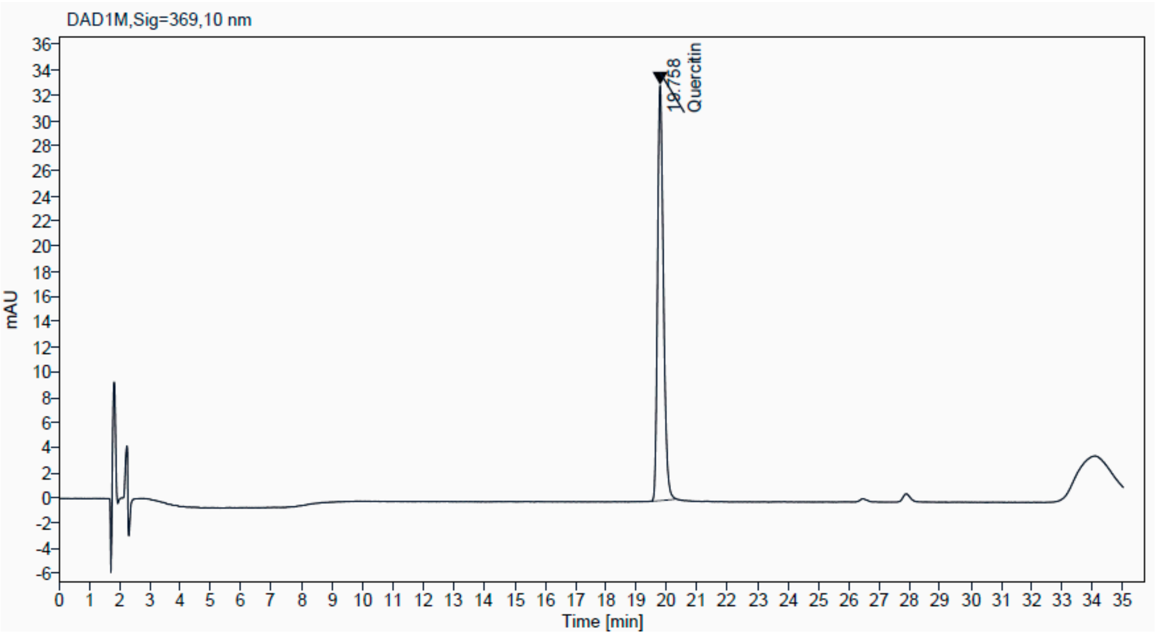

Figure S1. HPLC chromatogram for standard quercetin

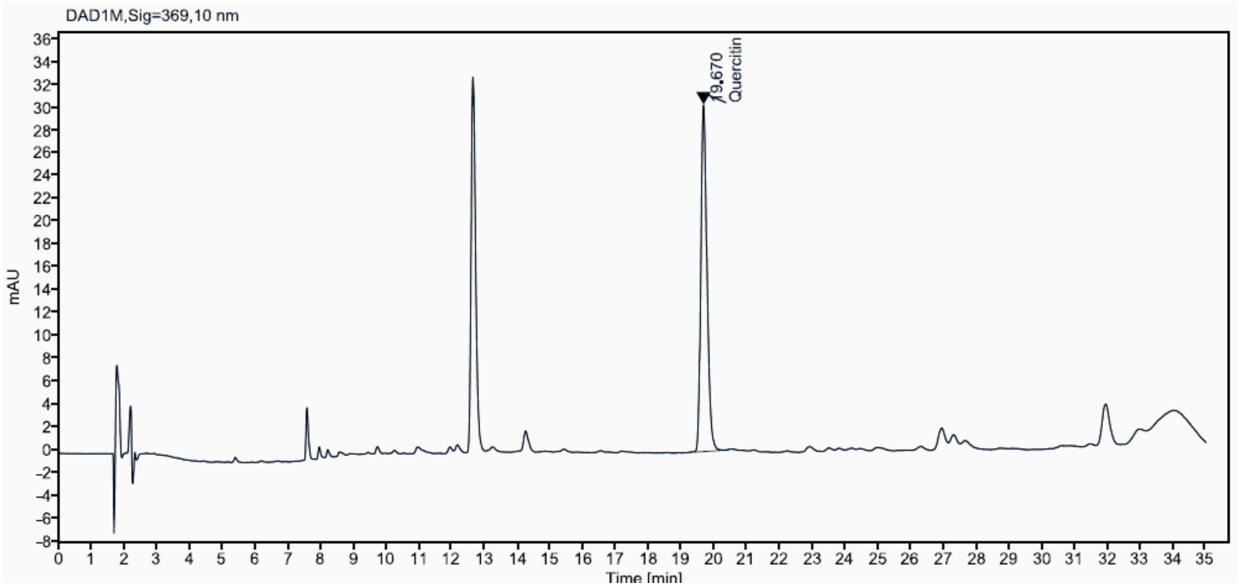

Figure S2. HPLC chromatogram of extracts obtained from yellow onion peels under optimum extraction conditions
